# Editorial: Collagen IV nephropathies: Alport syndrome and beyond

**DOI:** 10.3389/fmed.2022.1039949

**Published:** 2022-09-30

**Authors:** Dorin-Bogdan Borza, Dale R. Abrahamson, Oliver Gross, Judy Savige

**Affiliations:** ^1^Department of Microbiology, Immunology and Physiology, School of Medicine, Meharry Medical College, Nashville, TN, United States; ^2^Department of Cell Biology and Physiology and the Jared Grantham Kidney Institute, University of Kansas Medical Center, Kansas City, KS, United States; ^3^Department of Nephrology and Rheumatology, University Medical Center Goettingen, Goettingen, Germany; ^4^Department of Medicine (Melbourne Health and Northern Health), The University of Melbourne, Parkville, VIC, Australia

**Keywords:** glomerular basement membrane (GBM), collagen type IV, thin glomerular basement membrane disease, collagen IV nephropathies, COL4A3 mutation, COL4A4 mutations, COL4A5 mutations, Alport syndrome

Mammalian type IV collagen is a family of six chains that form three types of heterotrimeric molecules, which assemble into supramolecular networks in basement membranes. A network comprising α345(IV) collagen, produced by podocytes, is a major component of the glomerular basement membrane (GBM), important for maintaining the normal function of the glomerular filtration barrier (1). Pathogenic variants in the *COL4A3, COL4A4*, and *COL4A5* genes encoding the α3(IV), α4(IV), and α5(IV) collagen chains result in a spectrum of nephropathies with diverse clinical presentation.

The most severe forms are X-linked and autosomal recessive (AR) Alport syndrome, characterized by progressive kidney failure, hearing loss, and ocular abnormalities. Heterozygous pathogenic variants in the *COL4A3* and *COL4A4* genes result in autosomal dominant (AD) Alport syndrome, formerly known as thin membrane nephropathy, which represents the carrier state for AR Alport syndrome (2). Digenic Alport syndrome denotes pathogenic changes in two of the three Alport genes, such as *COL4A5* plus *COL4A3*; or *COL4A3* plus *COL4A4* (3). AD Alport syndrome is the commonest genetic kidney disease affecting about one in 100 individuals, while X-linked Alport syndrome affects about one in 2,000, and AR and digenic Alport syndrome are both much rarer (4).

This Research Topic presents a collection of articles describing developments in the area of collagen IV nephropathies, including but not limited to Alport syndrome.

The article by Savige et al. reviews genotype-phenotype correlations for pathogenic *COL4A3–COL4A5* variants. The variant features that determine disease severity are the same for *COL4A5, COL4A3*, and *COL4A4* in X-linked, AR, and AD Alport syndrome. Large rearrangements, truncating variants, and splice site changes generally result in more severe disease than missense variants. For missense variants, Gly substitutions result in more severe disease than non-Gly substitutions. Among these, Gly substitutions with bulkier residues (e.g., Arg, Glu, Asp, Trp, Val) are associated with more severe disease than substitutions with small residues (Ala, Ser). Understanding genotype-phenotype correlations in Alport syndrome is important because they help predict early onset kidney failure and extra-renal complications, and the need for therapy with renin-angiotensin-aldosterone blockade (5).

The article by Comić et al. also addresses the multifaceted phenotypic and genotypic spectrum of collagen IV nephropathies. This study illustrates the complex clinical and genetic picture of individuals with a type IV-collagen-related nephropathy, indicating the need for a refined nomenclature and teamwork between clinicians and geneticists.

The article by Cerkauskaite et al. reports the analysis by next generation sequencing in a cohort of Lithuanians with suspected Alport syndrome. Molecular testing of 171 individuals led to the detection of 99 individuals with 44 disease-causing variants, including 27 novel variants (nine in each of the *COL4A3, COL4A4*, and *COL4A5* genes).

Recent advances in genetic analysis highlight the importance of detecting splicing variants in *COL4A5*, which account for about 15% of all cases of X-linked Alport syndrome. Aberrant splicing results from both canonical and non-canonical splice site variants, the latter including deep intronic changes and substitution in exons. The article by Yamamura et al. reviews the contribution of *COL4A5* splicing variants to the pathogenesis of X-linked Alport syndrome, the latest diagnosis strategies, and the prospects for new therapeutic approaches.

One article by Deng et al. identifies two *COL4A3* variants initially presumed to be missense [p.(Leu1598Arg)] or synonymous [p.(Thr255Thr)], which instead were demonstrated to induce aberrant RNA splicing. These findings highlight the importance of transcript analysis of unclassified exonic sequence variants for better molecular diagnosis.

The second article by Deng et al. reports the detection of low-level somatic mosaic *COL4A5* splicing variant in an asymptomatic female, who gave birth to two boys with X-linked Alport syndrome caused by a hemizygous disease-causing *COL4A5* variant. Although the disease-causing variant was not detected in the mother's genomic DNA by Sanger sequencing, both wild type and very low-level mutant *COL4A5* were identified by droplet digital PCR. This illustrates that some cases of X-linked Alport syndrome attributed to presumed *de novo COL4A5* mutations are due to parental mosaicism.

Mutations in the *COL4A3–COL4A5* genes are the commonest cause of inherited kidney failure after polycystic kidney disease. No specific therapies for Alport syndrome exist yet. Chavez et al. provide an overview of novel therapeutic agents to arrest disease progression in Alport syndrome, currently under investigation. These include an oral Nrf2 activator, anti-miRNA-21 oligonucleotides, endothelin type A receptor inhibitors, inducers of cholesterol efflux, DDR1 inhibitors, osteopontin-blocking agents, as well as the drugs hydroxychloroquine, metformin, and paricalcitol. The review also discusses future therapeutic strategies such as chaperon therapy, genome editing, and stem cell therapy.

Cosgrove and Madison review the consequences of the altered GBM composition in Alport syndrome, with emphasis on the molecular and cellular mechanisms underlying the initiation and progression of Alport glomerular pathology. Specifically, they cite evidence for upregulation of endothelin-1 in glomerular endothelial cells, activation of endothelin A receptors and CDC42 in mesangial cells, and ectopic deposition of mesangial matrix proteins in Alport GBM.

The α345(IV) collagen is also the autoantigen targeted pathogenic anti-GBM autoantibodies, which bind to the GBM and alveolar basement membranes causing rapidly progressive glomerulonephritis and pulmonary hemorrhage (6). A case report by Sobotta et al. describes a patient with acute respiratory distress syndrome secondary to anti-GBM antibodies, who has recovered pulmonary function after treatment with eculizumab—a monoclonal antibody that binds to complement C5 and prevents its cleavage by C5 convertases, thereby inhibiting the activation of the terminal complement cascade.

In summary, the current Research Topic is a collection of nine original and review articles which describe genotype-phenotype correlations for all three Alport genes, report novel pathogenic variants (including uncommon types), review the mechanisms of initiation and progression of Alport glomerular pathology, and overview novel therapies for Alport syndrome. These articles highlight the most recent developments in these areas of research.

## Author contributions

D-BB wrote the original draft. DA, OG, and JS edited the manuscript. All authors reviewed and approved the final manuscript.

## Funding

D-BB was supported by a pilot grant funded by the grant U54 MD007593 from the National Institute on Minority Health and Health Disparities of the National Institutes of Health, and by award W81XWH-20-1-0698 from the Lupus Research Program of the US Department of Defense. OG was supported by the German Research Foundation DFG (GR 1852/6-1).

## Conflict of interest

The authors declare that the research was conducted in the absence of any commercial or financial relationships that could be construed as a potential conflict of interest.

## Publisher's note

All claims expressed in this article are solely those of the authors and do not necessarily represent those of their affiliated organizations, or those of the publisher, the editors and the reviewers. Any product that may be evaluated in this article, or claim that may be made by its manufacturer, is not guaranteed or endorsed by the publisher.

## References

[1] Abrahamson DR Hudson BG Stroganova L Borza DB St John PL. Cellular origins of type IV collagen networks in developing glomeruli. J Am Soc Nephrol. (2009) 20:1471–9. 10.1681/ASN.2008101086 19423686 PMC2709682

[2] Savige J Rana K Tonna S Buzza M Dagher H Wang YY. Thin basement membrane nephropathy. Kidney Int. (2003) 64:1169–78. 10.1046/j.1523-1755.2003.00234.x 12969134

[3] Savige J Renieri A Ars E Daga S Pinto AM Rothe H. Digenic Alport syndrome. Clin J Am Soc Nephrol. (2022). 10.2215/CJN.03120322. [Epub ahead of print]. 35675912 PMC9718039

[4] Gibson J Fieldhouse R Chan MMY Sadeghi-Alavijeh O Burnett L Izzi V. Prevalence estimates of predicted pathogenic COL4A3-COL4A5 variants in a population sequencing database and their Implications for Alport syndrome. J Am Soc Nephrol. (2021) 32:2273–90. 10.1681/ASN.2020071065 34400539 PMC8729840

[5] Savige J Gregory M Gross O Kashtan C Ding J Flinter F. Expert guidelines for the management of Alport syndrome and thin basement membrane nephropathy. J Am Soc Nephrol. (2013) 24:364–75. 10.1681/ASN.2012020148 23349312

[6] Borza DB Hudson BG. Molecular characterization of the target antigens of anti-glomerular basement membrane antibody disease. Springer Semin Immunopathol. (2003) 24:345–61. 10.1007/s00281-002-0103-1 12778332

